# Two new species of Comesomatidae (Nematoda, Araeolaimida) from the Rizhao coast of the Yellow Sea, China

**DOI:** 10.3897/zookeys.1275.164380

**Published:** 2026-03-31

**Authors:** Huiying Yu, Chunming Wang

**Affiliations:** 1 College of Agriculture and Biology, Liaocheng University, Liaocheng, 252059, China Liaocheng University Liaocheng China

**Keywords:** *
Comesoma
*, marine nematode, *
Paramesonchium
*

## Abstract

To study the diversity and taxonomy of marine nematodes on the Rizhao coast, undisturbed sediment samples have been systematically collected since 2022. Two new species of the family Comesomatidae, *Comesoma macramphida***sp. nov**. and *Paramesonchium tubulosupplementa***sp. nov**. are described. *Comesoma macramphida***sp. nov**. is characterized by having the outer labial sensilla setiform, the buccal cavity cup-shaped with a cuticularized projection, the amphidial fovea wide with 2.5 turns, spicules L-shaped with faint striation, the gubernaculum short with a small caudally directed apophysis, 10 or 11 papilliform precloacal supplements, and a conico-cylindrical tail with two long terminal setae. *Paramesonchium tubulosupplementa***sp. nov**. is characterized by having the cuticle ornamented with transverse punctations and lateral pores, four long cephalic setae, a wide buccal cavity with three ridges, a wide amphidial fovea with 2.5–2.75 turns, spicules curved with a cephalate proximal end, the gubernaculum with a dorso-caudal apophysis, 11 or 12 tubular precloacal supplements, and a conico-cylindrical tail with a short, cylindrical portion.

## Introduction

Marine nematodes represent the most abundant and taxonomically diverse group within the phylum Nematoda and are a dominant component of meiofaunal communities in marine sediments worldwide (Meldal et al. 2007; Bik et al. 2010). Despite their ecological significance and remarkable species richness, marine nematodes remain one of the most taxonomically challenging groups due to their small size, morphological convergence, and the paucity of molecular data available for phylogenetic analysis (Leduc et al. 2017; Hodda 2022).

The Yellow Sea, situated between China and the Korean Peninsula, represents a biogeographically important region for studies on marine biodiversity. As part of a comprehensive investigation into the meiofaunal diversity of this region, systematic sampling of undisturbed sediments was initiated along the Rizhao coast in May 2022. Preliminary analyses have revealed marine nematodes as the dominant meiobenthic taxon, with several new species already described and subjected to molecular phylogenetic analysis (Guo et al. 2023a, 2023b; Liang et al. 2024a, 2024b). These findings underscore the taxonomic richness of the region and highlight the need for continued systematic research.

The family Comesomatidae Filipjev, 1918, was established with *Comesoma* Bastian, 1865 as the type genus. The family has long presented taxonomic challenges due to the combined morphological structure. It exhibits a combination of features typically associated with different nematode orders: the punctate cuticle, spiral amphidial fovea, and tubular precloacal supplements are characteristic of Chromadorida, while the outstretched ovaries are more typical of Monhysterida (Jensen 1979; Lorenzen 1994; Fonseca and Bezerra 2014). This morphological complexity has resulted in ongoing debate regarding the phylogenetic placement of Comesomatidae. Currently, Comesomatidae is provisionally assigned to the order Araeolaimida based on morphological criteria (Lorenzen 1994; Fonseca and Bezerra 2014) and comprises three subfamilies: Comesomatinae Filipjev, 1918, Dorylaimopsinae De Coninck, 1965, and Sabatieriinae Filipjev, 1934 (Nemys Eds 2025). Within Comesomatidae, the genus *Comesoma* was established with the type species *C. vulgare* Bastian, 1865, currently comprised 13 valid species prior to this study. Similarly, the genus *Paramesonchium* Hopper, 1967, was established following the transfer of *Paramesonchium seriale* (Wieser, 1954) Hopper, 1967 from the genus *Laimella* Cobb, 1920, includes only three valid species before the present study.

This paper describes two new species of Comesomatidae recovered from the Rizhao coast: *Comesoma macramphida* sp. nov. and *Paramesonchium tubulosupplementa* sp. nov. Detailed morphological descriptions and illustrations are provided to improve our understanding of marine nematode diversity in the Yellow Sea, China.

## Materials and methods

Undisturbed sediment samples were collected from the intertidal zone of Rizhao coast using a syringe (2.9 cm inner diameter) to a depth of 8 cm. Each core was vertically subdivided into two depth intervals: 0–2 and 2–8 cm. Samples were immediately fixed with 10% formalin in seawater. In the laboratory, nematodes were stained with rose bengal, separated from the sediment samples, and slide-mounted as our previous article (Guo et al. 2023a).

Morphological examination was created using a differential interference contrast microscope (Axiscope-5, Zeiss, Germany). Line drawings were prepared using Procreate software on an iPad (Apple, USA), and photomicrographs were captured using ZEN software (Zeiss Corporation, Germany). Holotype and paratype specimens were deposited at the College of Agriculture and Biology, Liaocheng University.

## Results and discussion

### Taxonomy

**Order Araeolaimida De Coninck & Schuurmans Stekhoven, 1933**

**Family Comesomatidae Filipjev, 1918**

#### Genus Comesoma

Taxon classificationAnimaliaAraeolaimidaComesomatidae

Bastian, 1865

6EA662EA-5F08-5FC8-87FB-7E22E34B7B23

##### Diagnosis

(revised based on Fu et al. 2022). Cuticle characterised by transverse punctations without lateral differentiation. Anterior sensilla in three distinctly separated crowns; outer labial lateral setae usually shorter than the cephalic setae. Subcephalic setae present in one or more crowns, just posterior to cephalic setae. Buccal cavity cup-shaped, and posterior portion collapsed, weakly sclerotised and with three blunt or thorn-like projections at the border to anterior portion. Spicules long and slender. Gubernaculum plate-like or indistinct.

##### Remarks.

The genus *Comesoma* was first established with *C. vulgare* and *C. profundi* Bastian, 1865 from Falmouth, England. The genus diagnostic characteristics include: cup-shaped buccal cavity without teeth, spiral-shaped amphidial fovea, cuticle with transverse rows of fine punctations, lateral differentiation only a bit more coarsely punctuated, spicules usually elongated, and precloacal supplements usually present (Bastian 1865). Later, *C. heterura* Cobb, 1898 (accepted as *Sabatieria heterura* (Cobb, 1898) Filipjev 1918), *C. jubata* Cobb, 1898 (accepted as *Setosabatieria hilarula*), and *C. simile* Cobb, 1898 (based on female specimen) were described. Subsequently, *C. stenocephalum* Filipjev, 1918, *C. dubium* Filipjev, 1918 (accepted as *Paracomesoma dubium* (Filipjev, 1918) Schuurmans Stekhoven 1950), *C. minimum* Chitwood, 1937, *C. punctata* Schuurmans Stekhoven, 1950, *C. arenae* Gerlach, 1956, *C. sipho* Gerlach, 1956 (accepted as *Paracomesoma sipho* (Gerlach, 1956) Jensen and Gerlach 1977 ), *C. bermudense* Jensen & Gerlach, 1977, *C. solum* Pastor de Ward, 1984, and *C. hermani* Chen & Vincx, 1998 were described. Wieser (1954) synonymised *Sabatieria tenuispiculum* (Ditlevsen, 1921) with *C. tenuispiculum*. *Comesoma punctata* was transferred to *Metacomesoma* by Wieser (1954), but Jensen (1979) regarded this taxon as a species inquirenda due to the poor description of anterior sensilla arrangement. Recently, with the description of *C. sinica* Fu, Cai, Leduc & Lin, 2022 and *C. quattuordecimsupplementata* Xiao & Guo, 2023, 13 species are considered as valid.

According to Jensen (1979), the cephalic organs in the Comesomatidae are usually arranged in three distinct crowns with the inner labial and outer labial sensilla typically papilliform. When the six outer labial sensilla are setiform, the two lateral setae are sometimes longer than the other four setae and situated at the second and third crowns. The four cephalic setae are generally longer than the outer labial setae.

#### Comesoma macramphida sp. nov.

Taxon classificationAnimaliaAraeolaimidaComesomatidae

EE40F3F1-1625-5384-B6BD-7EDB3C4BF4F9

https://zoobank.org/26C5F69A-BAC9-44C5-9F96-A4A5BB662282

**Figure 1.**
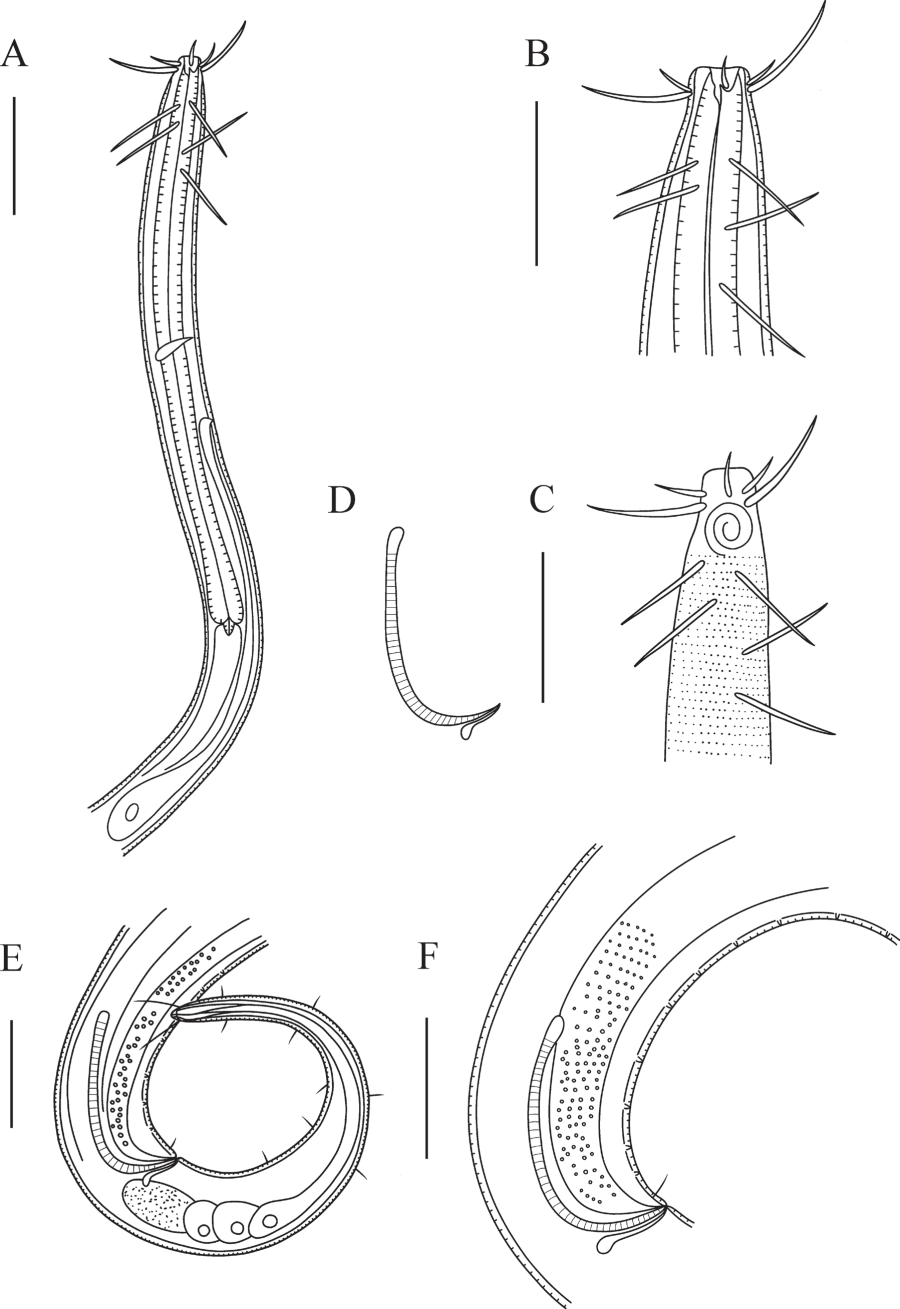
*Comesoma macramphida* sp. nov., holotype. **A**. Lateral view of male anterior region; **B**. Lateral view of male anterior region showing buccal cavity; **C**. Lateral view of male anterior region showing cuticle and amphidial fovea; **D**. Lateral view of spicules and gubernaculum; **E**. Lateral view of male posterior body; **F**. Lateral view of male posterior region showing precloacal supplements. Scale bars: 50 µm (**A**); 30 µm (**B, C, E, F**).

**Figure 2.**
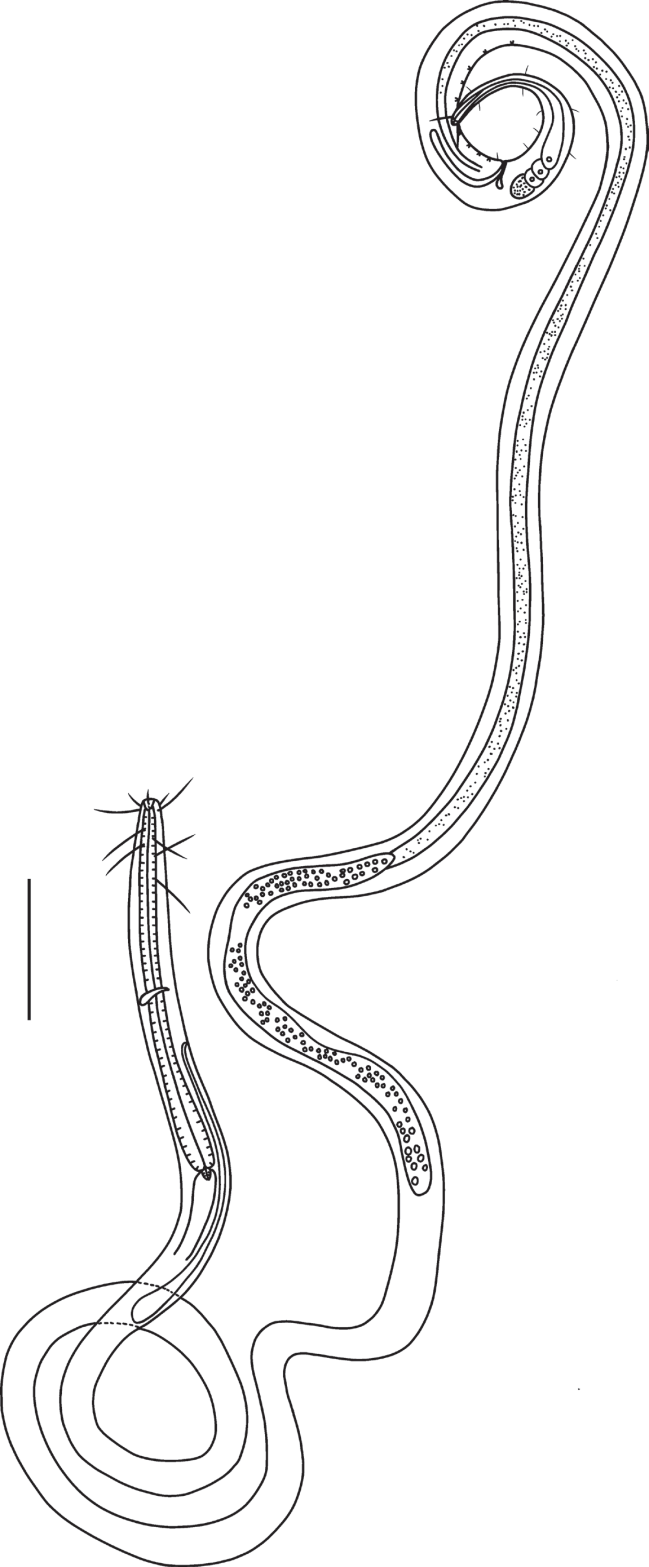
*Comesoma macramphida* sp. nov. Lateral view of male whole body (holotype). Scale bar: 80 µm.

**Figure 3.**
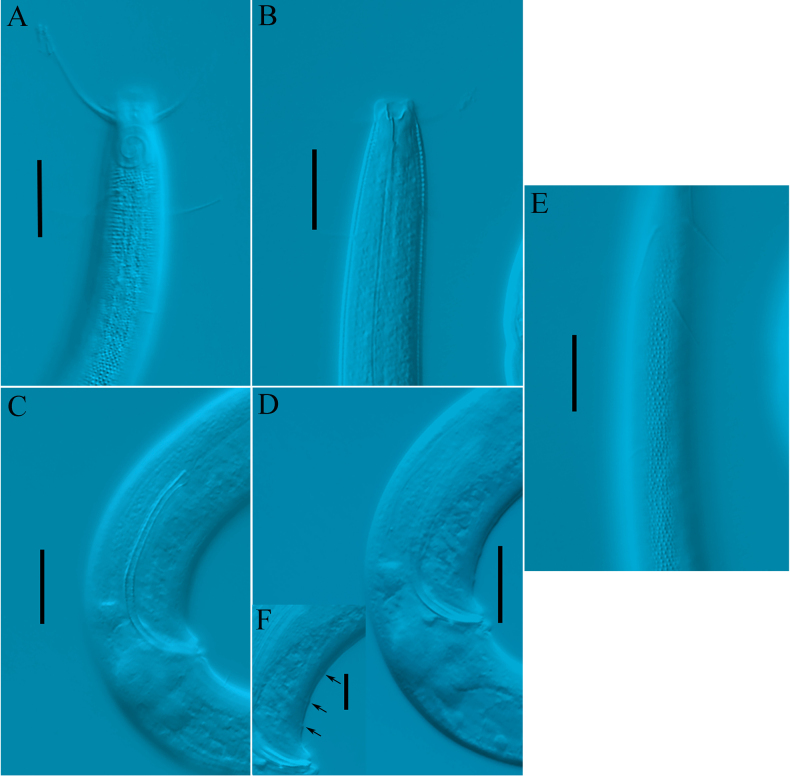
*Comesoma macramphida* sp. nov., holotype. **A**. Lateral view of male anterior region showing amphidial fovea and cephalic setae; **B**. Lateral view of male anterior region showing buccal cavity; **C**. Lateral view of male posterior region showing spicules; **D**. Lateral view of male posterior body, showing gubernaculum; **E**. Lateral view of male anterior region, showing lateral cuticle region; **F**. Lateral view of male posterior body, showing precloacal supplements (arrows). Scale bars: 20 µm (**A–E**); 10 µm (**F**).

Figs 1, 2, 3, Table 1

##### Diagnosis.

*Comesoma macramphida* sp. nov. is characterised by the following: outer labial sensilla setiform, buccal cavity cup-shaped with a cuticularized projection, amphidial fovea wide (77–92% of the body diameter) and consisting of 2.5 turns, spicules L-shaped and with faint striation; gubernaculum short and boat-shaped, with a small caudally directed apophysis, 10 or 11 papilliform precloacal supplements and tail conico-cylindrical, with two long, terminal setae.

##### Type material.

Three males were measured and studied. ***Holotype***: • ♂ on slide 22WPK10-3-13; ***paratype*** 1: • ♂ on slide 22WPK10-2-15; ***paratype*** 2: • ♂ on slide 22WPK10-3-2.

##### Type locality and habitat.

Rizhao coast, Shandong Province, China (35°26'N, 119°34'E). Specimens collected from sandy sediment at a depth of 0–2 cm; collected May 2022 by Wen Guo.

##### Measurements.

All morphometric measurement data are given in Table 1.

**Table 1.** Individual measurements of *Comesoma macramphida* sp. nov. (in µm, except ratio).

Characters	Holotype male	Paratype male #1	Paratype male #2
Total body length	2776	2753	2933
Maximum body diameter	30	35	28
Head diameter	11	11	10
Length of outer labial setae	6	6	6
Length of cephalic setae	24	23	30
Buccal cavity depth	4	4	4
Amphidial fovea width	10	12	11
Amphidial fovea turns	2.5	2.5	2.5
Amphidial fovea from anterior	5	5	5
Body diameter at amphidial fovea	13	13	14
Nerve ring from anterior	107	119	115
Body diameter at nerve ring	23	21	22
Pharynx length	219	228	228
Body diameter at pharynx	24	24	22
Anal body diameter	29	25	28
Spicule length along arc	64	59	62
Gubernaculum length	14	14	11
Precloacal supplements	10	11	10
Tail length	157	164	156
a	92.5	78.7	104.8
b	12.7	12.1	12.9
c	17.7	16.8	18.8
c'	5.4	6.6	5.6

##### Description.

**Males**. Body cylindrical, slender, and long; anterior end truncated, and posterior end tapered. Cuticle punctated; punctations arranged in transverse rows throughout body; lateral differentiation with irregularly spaced larger punctations. Cephalic sensilla arranged in three circles of 6+6+4. Inner labial sensilla 6, papilliform. Outer labial sensilla 6, setiform, 0.55–0.6 head diameters in length. Cephalic setae 4, long, 2.1–3.0 head diameters in length. Cervical setae 8, 11–12 μm in length; somatic setae present in pharynx and caudal regions, 2–4 μm in length. Amphidial fovea spiral, with 2.5 turns, and wide, 77–92% of body diameter, located immediately posterior to cephalic setae. Ocelli absent. Buccal cavity cup-shaped, 4 μm in depth, with subventral cuticularized projection at posterior. Pharynx muscular, cylindrical, and slightly expanded at posterior end but not forming true posterior bulb. Cardia conical, surrounded by intestine, and 7 μm in length. Nerve ring located in approximate middle of pharynx region, 48.9–52.2% of pharynx length. Secretory excretory system present; secretory excretory pore 142–146 μm from anterior end. Renette cell oval, 31–41 μm in length, 14–20 μm in width, and 68–76 μm from pharynx posterior end.

The reproductive system diorchic, with opposed and outstretched testes. Anterior testis to left of intestine and posterior testis to right. Spicules paired, L-shaped, with faint striation, 2.21–2.36 cloacal body diameters long. Gubernaculum boat-shaped, short, and parallel to distal end of spicules, and with a very small caudally directed apophysis. Precloacal supplements 10 or 11, papilliform; anteriormost precloacal supplement 133–171 μm from cloacal opening and posteriormost 10–14 μm from cloacal opening, respectively; distance slightly widening from posterior to anterior. Tail conico-cylindrical, 5.4–6.6 anal body diameters long, with cylindrical part about 1/3 of total length. Two terminal setae present, 16–17 μm in length. Four caudal glands in line with separate outlet.

Female not observed.

##### Etymology.

The species epithet *macramphida* is derived from Latin *macro* (large) and *amphida* (amphidial) and refers to the large size of amphidial fovea.

##### Differential diagnosis and discussion.

*Comesoma macramphida* sp. nov. can be differentiated from other species by the combination of having spicules shorter than 100 μm and six outer labial sensilla setiform. The new species is similar to *C. bermudense*, *C. hermani*, *C. minimum*, *C. quattuordecimsupplementata*, *C. sinica*, *C. stenocephalum*, and *C. tenuispiculum* in amphidial fovea turns. This new species differs from *C. bermudense* in the length of the outer labial setae (6 μm vs 2 μm), ornamentation and length of spicules (59–64 μm, with faint striation vs 150–194 μm, without striation), gubernaculum length (11–14 μm vs 25 μm), and number of precloacal supplements (10–11 vs 18–22). *Comesoma macramphida* sp. nov. differs from *C. hermani* in the length of the body (2753–2933 μm vs 1694–2108 μm), length of the cephalic setae (23–30 μm vs 13 μm), spicule length (59–64 μm vs 169–184 μm), and length of the gubernaculum (11–14 μm vs 20–25 um). *Comesoma macramphida* sp. nov. differs from *C. minimum* in the length of the body (2753–2933 μm vs 1370–1520 μm), length of the cephalic setae (2.1–3.0 head diameters vs 1.1 head diameters) and spicule length (2.2–2.4 anal body diameters vs 6.3 anal body diameters) (Xiao and Guo 2023). *Comesoma macramphida* sp. nov. differs from *C. quattuordecimsupplementata* in the shape of the buccal cavity (cup-shaped, wide and with cuticularized projection vs cup-shaped and small, without a projection), length of cephalic setae (23–30 μm vs 9–10 μm), spicule length (59–64 μm vs 120–128 μm), and number of precloacal supplements (10–11 vs 14). *Comesoma macramphida* sp. nov. differs from *C. sinica* in spicule length (59–64 μm vs 166–179 μm) and number of precloacal supplements (10–11 vs >20); the new species differs from *C. stenocephalum* in the length of the body (2753–2933 μm vs 4600 μm), depth of the buccal cavity (4 μm vs 8 μm), spicule length (59–64 μm vs 165 μm), and length of the gubernaculum (11–14 μm vs 40 μm). *Comesoma macramphida* sp. nov. differs from *C. tenuispiculum* in the length of the body (2753–2933 μm vs 1600 μm), the length of the cephalic setae (2.1–3.0 head diameters vs 1.5 head diameters), spicule length (59–64 μm vs 112 μm), and tail length (5.4–6.6 anal body diameters vs 3.5 anal body diameters, measured according to Filipjev 1918).

#### Genus Paramesonchium

Taxon classificationAnimaliaAraeolaimidaComesomatidae

Hopper, 1967

683425B9-6236-5767-BC23-5FB15734370D

##### Revised diagnosis

**(based on Fonseca and Bezerra 2014)**. Anterior sensilla in three circles of 6+6+4. Cuticle laterally differentiated, with transverse or longitudinal rows of punctations; lateral pore complex either absent or present. Posterior portion of buccal cavity conical, with three ridges; each ridge ending in an acute projection at border to anterior portion. Spicules curved. Gubernaculum with dorso-caudally directed apophysis. Precloacal supplements present, setiform or tubular.

##### Remarks.

The genus *Paramesonchium* was established with the *P. seriale* as its type species, which was transferred from genus *Laimella* based on the presence of longitudinal rows of coarse punctations on the cuticle and buccal cavity conoid shaped. Subsequently, additional species were described: *P. belgicum* Jensen, 1976, *P. angelae* (Inglis, 1968), and *P. mombasi* Muthumbi, Soetaert & Vincx, 1997. Zhang (1992) later transferred *P. angelae* to the genus *Dorylaimopsis* Ditlevsen, 1918.

The morphological characteristics of *Paramesonchium* exhibit considerable variation. Notably, *P. seriale* was described based on a female specimen and was characterised by being laterally differentiated and having the buccal cavity with three acute tips; *P. belgicum* was characterised by lacking cuticle differentiation and having a lateral pore complex present, a buccal cavity with three acutely pointed lips, and setiform precloacal supplements each with a duct. *Paramesonchium mombasi* was characterised by having a cuticle with differentiation, a buccal cavity lacking teeth, and setiform precloacal supplements. Based on current taxonomic understanding, *Paramesonchium* should encompass species in which the cuticle either has or lacks lateral differentiation, the lateral pore complex is present, and precloacal supplements are either setiform or tubular (as demonstrated below).

#### Paramesonchium tubulosupplementa sp. nov.

Taxon classificationAnimaliaAraeolaimidaComesomatidae

DCE007C9-28DB-5264-92FF-048D1610AC98

https://zoobank.org/6a22b88c-85c7-497f-8016-f02916e9ee58

Figs 4, 5, Table 2

##### Diagnosis.

*Paramesonchium tubulosupplementa* sp. nov. is characterised by the following: body length of 2002–2056 µm; cephalic setae four, 2.5–3.0 head diameters long; cuticle with transverse punctations and a pore complex; amphidial fovea wide, spiral, and with 2.5–2.75 turns; spicules short, curved, and with proximal end cephalated; gubernaculum with dorso-caudal apophysis; tubular precloacal supplements 11 or 12; tail conico-cylindrical, with cylindrical portion short.

**Figure 4.**
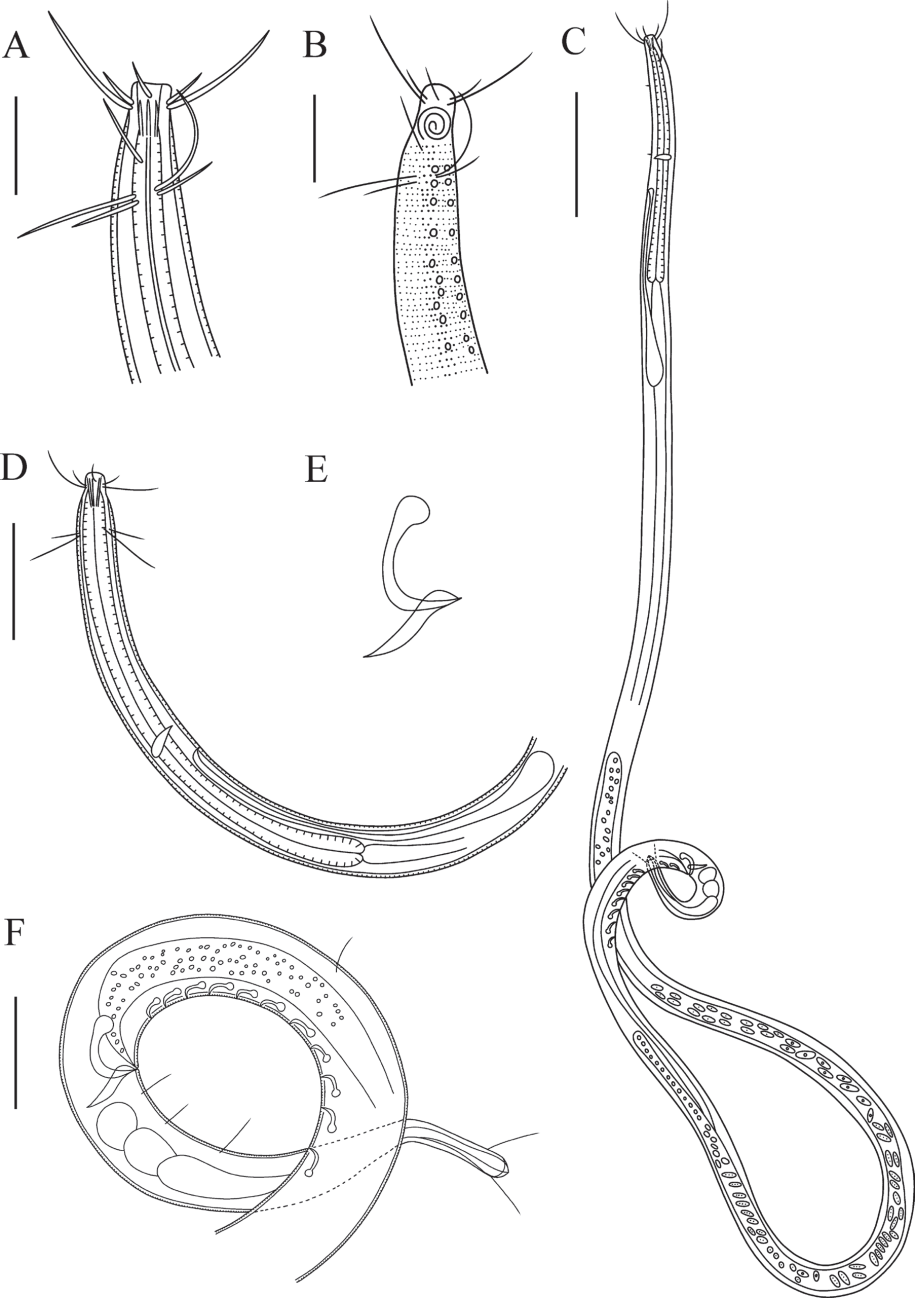
*Paramesonchium tubulosupplementa* sp. nov. **A**. Lateral view of male anterior region showing buccal cavity and anterior sensilla (holotype); **B**. Lateral view of male anterior region showing cuticle, amphidial fovea and anterior sensilla (holotype); **C**. Lateral view of male whole body (22SHT5-3-4); **D**. Lateral view of male anterior region (22RZDT4-3-3); **E**. Lateral view of spicules and gubernaculum (22RZDT4-3-3); **F**. Lateral view of male posterior body (22RZDT4-3-3). Scale bars: 20 µm (**A, B**); 80 µm (**C**); 50 µm (**D**); 30 µm (**F**).

**Figure 5.**
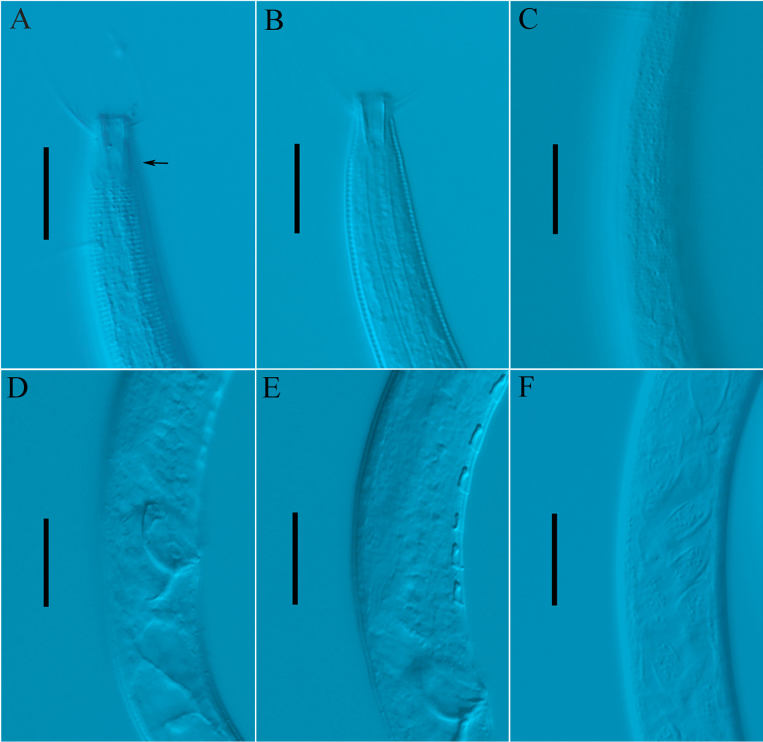
*Paramesonchium tubulosupplementa* sp. nov. **A**. Lateral view of male anterior region, arrow for amphidial fovea (holotype); **B**. Lateral view of male anterior region showing buccal cavity (holotype); **C**. Lateral view of male anterior region showing cuticle (holotype); **D**. Lateral view of male posterior body, showing spicules and gubernaculum (holotype); **E**. Lateral view of male posterior region, showing precloacal supplements (holotype); **F**. Lateral view of male posterior body, showing sperms (22RZDT4-3-3). Scale bars: 20 µm.

##### Type material.

Three males were measured and examined. ***Holotype***: • ♂, on slide 22LJW3-3-18; ***paratype*** 1: • ♂, on slide 22SHT5-3-4; ***paratype*** 2: • ♂, on slide 22RZDT4-3-3.

##### Type locality and habitat.

Rizhao coast, Shandong Province, China, (35°18'N, 119°31'E). Specimens were collected from sandy sediment at a depth of 0–2 cm; collected May 2022 by Wen Guo.

##### Measurements.

All morphometric measurement data are given in Table 2.

**Table 2.** Individual measurements of *Paramesonchium tubulosupplementa* sp. nov. (in µm, except ratio).

Characters	Holotype male	Paratype male #1	Paratype male #2
Total body length	2030	2056	2002
Maximum body diameter	25	27	24
Head diameter	8	9	8
Length of outer labial setae	6	7	6
Length of cephalic setae	24	27	20
Buccal cavity depth	13	14	13
Amphidial width	9	10	10
Amphidial turns	2.75	2.75	2.5
Amphidial fovea from anterior	5	6	6
Body diameter at amphidial fovea	11	11	11
Nerve ring from anterior	99	98	97
Body diameter at nerve ring	20	21	20
Pharynx length	253	237	230
Body diameter at the base of pharynx	22	23	21
Anal body diameter	23	23	21
Spicule length along arc	24	23	25
Gubernaculum length	12	10	12
Precloacal supplements	11	12	11
Tail length	124	127	127
a	81.2	76.1	83.4
b	8.0	8.7	8.7
c	16.4	16.2	15.8
c'	5.4	5.5	6.0

##### Description.

**Males**. Body cylindrical, long, and slender; anterior end truncated, and posterior end tapered. Cuticle with transverse rows of punctations throughout body; two longitudinal cuticle pore complexes present in pharynx region. Cephalic sensilla in three circles of 6+6+4. Inner labial sensilla 6, papilliform. Outer labial sensilla 6, setiform, 0.75–0.78 head diameters in length. Cephalic setae 4, long, 2.5–3.0 head diameters in length; two pairs of sublateral cervical setae, 27–28 μm and 6–11 μm in length, respectively. Somatic setae scarcely present, mainly in caudal region, 7 μm in length. Amphidial fovea faintly spiral, in 2.5–2.75 turns, wide, 81.8–90.9% of body diameter, and anterior end immediately posterior to cephalic setae. Ocelli absent. Buccal cavity wide, funnel-shaped, with three ridges, and 13–14 μm in depth. Pharynx muscular, cylindrical, and posterior end slightly expanded without forming a terminal bulb. Cardia inconspicuous. Nerve ring slightly anterior to middle pharynx region, 39.1–42.2% of pharynx length. Secretory excretory cell positioned posterior to pharynx; secretory excretory pore 134–148 μm from anterior end. Renette cell oval, 29–40 μm in length, 15–20 μm in width, and 46–71 μm from posterior end of pharynx.

Reproductive system diorchic, with opposed and outstretched testes. Anterior testis to left of intestine, and posterior to right. Sperm oval, 14 μm in length and 7 μm in width. Spicules paired, short, obviously curved, 1.0–1.2 cloacal body diameters in length, and with proximal end cephalated and distal end tapered. Gubernaculum short, with a dorso-caudally curved apophysis. Tubular precloacal supplements 11 or 12, 6–7 μm in length; anteriormost 100–140 μm and posteriormost 12–21 μm from cloacal opening, respectively; distance between supplements gradually increases from posterior to anterior. Tail conico-cylindrical, 5.4–6.0 cloacal body diameters in length; cylindrical portion 16.2–23.6% of total length of tail and with a slightly swollen tip. Caudal glands 3, with separate outlets. Two long setae at tail tip, 17–18 μm in length.

Female not observed.

##### Etymology.

The species epithet *tubulosupplementa* is derived from Latin *tubulo* (tubular) and *supplementa* (supplement) and refers to precloacal supplements tubular.

##### Differential diagnosis and discussion.

Currently, only three species have been described within genus *Paramesonchium*: *P. belgicum*, *P. mombasi*, and *P. seriale*. The new species differs from *P. belgicum* in the length of the cephalic setae (20–27 μm vs 36 μm), absence of denticles in the buccal cavity (a crown of denticles present in *P. belgicum*), the number of turns of the amphidial fovea (2.5–2.75 vs 3.75), spicule length (23–25 μm vs 38 μm), number and shape of precloacal supplements (11–12, tubular vs 5, setiform), and length of the cylindrical portion of the tail (16.2–23.6% vs 67%). *Paramesonchium tubulosupplementa* sp. nov. differs from *P. mombasi* in the depth of the buccal cavity (13–14 μm vs 7–9 μm), length and shape of the spicules (23–25 μm, proximal end cephalated vs 17 μm, proximal end not cephalated), number and shape of the precloacal supplements (11–12, tubular vs 6–7, setiform), and length of the cylindrical portion of the tail (16.2–23.6% vs 57%, measured according to Muthumbi et al. 1997). *Paramesonchium tubulosupplementa* sp. nov. differs from *P. seriale* in the number of turns of the amphidial fovea (2.5–2.75 vs 3), absence of cuticular ornamentation (present in *P. seriale*), and length of the cylindrical portion of the tail (16.2–23.6% vs 67%).

## Supplementary Material

XML Treatment for Comesoma

XML Treatment for Comesoma macramphida

XML Treatment for Paramesonchium

XML Treatment for Paramesonchium tubulosupplementa

## Additional information

### Use of AI

No use of AI was reported.

### Author ORCIDs

Chunming Wang https://orcid.org/0000-0003-3630-0921

### Data availability

All of the data that support the findings of this study are available in the main text.
